# Rim Enhancement after Technically Successful Transarterial Chemoembolization in Hepatocellular Carcinoma: A Potential Mimic of Incomplete Embolization or Reactive Hyperemia?

**DOI:** 10.3390/tomography8020094

**Published:** 2022-04-15

**Authors:** Kaspar Ekert, Christopher Kloth, Konstantin Nikolaou, Gerd Grözinger, Marius Horger, Wolfgang Thaiss

**Affiliations:** 1 Department of Diagnostic and Interventional Radiology, Eberhard-Karls-University, Hoppe-Seyler-Str. 3, 72076 Tuebingen, Germany; kaspar.ekert@uniklinik-ulm.de (K.E.); konstantin.nikolaou@med.uni-tuebingen.de (K.N.); gerd.groezinger@med.uni-tuebingen.de (G.G.); marius.horger@med.uni-tuebingen.de (M.H.); 2 Department of Nuclear Medicine, Ulm University Hospital, Albert-Einstein-Allee 23, 89081 Ulm, Germany; 3 Department of Diagnostic and Interventional Radiology, Ulm University Hospital, Albert-Einstein-Allee 23, 89081 Ulm, Germany; christopher.kloth@uniklinik-ulm.de

**Keywords:** carcinoma, hepatocellular, liver, 4D computed tomography, perfusion imaging, embolization, therapeutic

## Abstract

Contrast enhancement at the margins/rim of embolization areas in hepatocellular-carcinoma (HCC) lesions treated with transarterial chemoembolization (TACE) might be an early prognostic indicator for HCC recurrence. The aim of this study was to evaluate the predictive value of rim perfusion for TACE recurrence as determined by perfusion CT (PCT). A total of 52 patients (65.6 ± 9.3 years) underwent PCT directly before, immediately after (within 48 h) and at follow-up (95.3 ± 12.5 days) after TACE. Arterial-liver perfusion (ALP), portal-venous perfusion (PVP) and hepatic-perfusion index (HPI) were evaluated in normal liver parenchyma, and on the embolization rim as well as the tumor bed. A total of 42 lesions were successfully treated, and PCT measurements showed no residually vascularized tumor areas. Embolization was not entirely successful in 10 patients with remaining arterialized focal nodular areas (ALP 34.7 ± 10.1 vs. 4.4 ± 5.3 mL/100 mL/min, *p* < 0.0001). Perfusion values at the TACE rim were lower in responders compared to normal adjacent liver parenchyma and edges of incompletely embolized tumors (ALP liver 16.3 ± 10.1 mL/100 mL/min, rim responder 8.8 ± 8.7 mL/100 mL/min, rim non-responder 23.4 ± 8.6 mL/100 mL/min, *p* = 0.005). At follow-up, local tumor relapse was observed in 17/42, and 15/42 showed no recurrence (ALP 39.1 ± 10.1 mL/100 mL/min vs. 10.0 ± 7.4 mL/100 mL/min, *p* = 0.0008); four patients had de novo disseminated disease and six patients were lost in follow-up. Rim perfusion was lower compared to adjacent recurring HCC and not different between groups. HCC lesions showed no rim perfusion after TACE, neither immediately after nor at follow-up at three months, both for mid-term responders and mid-term relapsing HCCs, indicating that rim enhancement is not a sign of reactive hyperemia and not predictive of early HCC recurrence.

## 1. Introduction

Improving diagnostics in patients with hepatocellular carcinoma (HCC) demands accurate evaluation of therapy response. Transarterial chemoembolization (TACE) is a possible management of unresectable intermediate-stage HCC according to Barcelona Clinic for Liver Cancer’s staging (BCLC) [1] and has a central role as an effective treatment according to the European Association for the Study of the Liver (EASL) and the European Organization for Research and Treatment of Cancer (EORTC) guidelines [2], being one of the first therapeutic approaches to primary unresectable HCC in several countries [3]. A well-recognized imaging phenomenon after local therapies such as TACE and radiofrequency ablation (RFA) is a rim or ring-like enhancement at the border of the therapy, which is most prominently visualized in arterial-phase imaging in CT and MRI [4,5]. This common phenomenon presumably represents reactive hyperemia at the margins of the embolized tumor. It is still unclear whether this represents a reactive phenomenon or a potential mimic of residual-tumor tissue and has also been described in association with other local therapy regimens including radiofrequency ablation (RFA) and selective internal radiation therapy (SIRT) [6,7,8,9]. Depending on the type of interventional therapy, this feature should occur within few weeks, or several months for RFA [7,8]. Currently, immediate post-TACE evaluation is rarely performed and therefore data with respect to direct post-TACE effects are only known from conventional digital subtraction angiography, which is known to have limited validity in determining therapeutic efficacy [10,11,12,13,14,15,16]. Perfusion CT may offer a robust imaging technique that is less prone to artifacts and capable of detecting subtle changes in tumor vascularization and peri-tumoral liver parenchyma induced by these therapies [17,18,19]. In comparison, multi-slice three-phase contrast-enhanced CT consists of multiple repetitive CT scans and thus illustrates the tumor vascularization represented by several arterial as well as mixed arterial–portal-venous enhancement phases that allow for accurate perfusion quantification. The use of dedicated pharmacokinetic models enables separate calculations of hepatic arterial, portal-venous liver and tumor blood supply, thereby enabling differentiation between the two with increased accuracy in detecting residual tumors, e.g., after TACE [20]. Aided by the HCC characteristics from baseline (pre-TACE), perfusion CT might help to better understand the immediate post-TACE status.

The purpose of this study was therefore to identify the potential occurrence of increased rim enhancement very early after TACE and in mid-term follow-ups, using qualitative and quantitative perfusion-CT-image analysis to assess the role of peripheral changes as a predictor of mid-term response to TACE or as a sign of perifocal hyperemia.

## 2. Materials and Methods

### 2.1. Study Population

A total of 52 patients (65.6 ± 9.3 years, range 37–80 years, 12 female) with HCC were treated at our institution and referred to TACE therapy after the interdisciplinary consensus of a dedicated tumor board between January 2010 and December 2015. Two independent imaging modalities (contrast-enhanced CT, MRI, or ultrasound) meeting the diagnostic criteria in accordance with the EASL guideline [2,21] had to be presented for the diagnosis of HCC. In five cases with inconclusive imaging, the diagnosis was proven by histology. The therapeutic regimen with the indication for TACE was approved by the local tumor board in accordance with the current EASL guideline [21], excluding patients with severe hepatic decompensation and tumor burden > 50% of total liver tissue, macrovascular invasion, or portal-vein thrombosis.

Patients underwent PCT before, immediately (within 48 h) after TACE and at follow-up scheduled after three months (95.3 ± 12.5 days after therapy). Patients were enrolled prospectively. Informed consent from every participant was obtained and the study was approved by the local ethical committee.

Residual HCC after TACE was defined as measurable residual contrast enhancing tumor tissue. Recurrence at follow-up was defined as newly measurable tissue enhancement in the TACE region evaluated using the Modified Response Evaluation Criteria in Solid Tumors (mRECIST) and the PCT results by radiologists with experience in reading oncological imaging and interventional radiology. The cases were interdisciplinarily re-evaluated for defining the following therapeutic regimen.

### 2.2. CT Protocol

A 128-row and a 256-row CT scanner (Somatom Definition AS+, Definition Flash, Siemens Healthcare, Forchheim, Germany) were used with an initial low-dose non-enhanced CT (60 mAs, 100 kVp, 5 mm slice thickness) to plan the perfusion study. Adaptive spiral-scanning technique with 80 kVp, 100–120 mAs, 64 × 0.6 mm collimation was used. Scan range up to 7 cm coverage with a scan time of 40 s and a resolution in time of 1.5 s per spiral dataset was used. A test bolus using 7 mL contrast agent was used for assessment of the optimal delay time before perfusion start. A dual-head pump injector (Medtron, Saarbruecken, Germany) was used for the administration of 50 mL Ultravist 370 (Bayer, Leverkusen, Germany) with a flow rate of 5 mL/s. Examinations resulted in a mean dose-length product of 478.9 mGy cm.

### 2.3. DCE-CT Analysis

**Figure 1:**
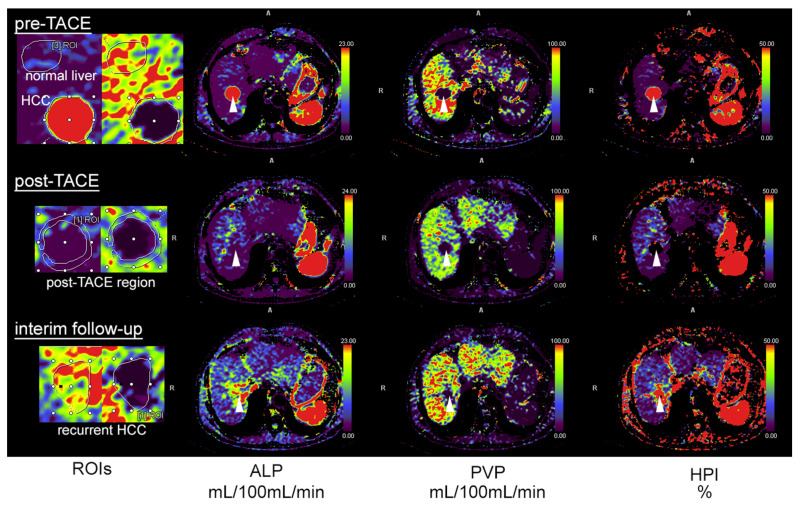
A 78-year-old patient with HCC and transarterial chemoembolization (TACE). Pre-TACE images (top row) show increased arterial-liver perfusion (ALP, second column, white arrowhead), reduced portal-venous perfusion (PVP, third column) and increased hepatic-perfusion index (HPI, right) compared to normal liver tissue. Post-TACE images (middle row) demonstrate a complete loss of lesion ALP, PVP and HPI, indicative of complete embolization. The follow-up after three months shows recurrent ALP at the site of embolization, highly suggestive of local recurrence. The first column illustrates how regions of interest (ROIs) were drawn with delineation of the HCC and adjacent normal liver tissue (**top**), the rim of the post-TACE region (**middle**) and at the site of embolization in the follow-up examination (**bottom**).

Syngo.via body perfusion (version VB10B, Siemens Healthineers, Forchheim, Germany) was used for subsequent motion correction, noise reduction and threshold-based exclusion of bone, fat and air [22]. A volume of interest (VOI) was drawn at the site of maximal arterial-liver perfusion of the tumors as well as at the tumor margins after TACE and apart from the tumor in the normal liver parenchyma avoiding larger vessels (Figure 1). Residual tumor at the location of TACE or recurrent enhancement at follow-up was measured accordingly. The largest lesion was evaluated in all cases.

The following perfusion parameters were quantified: arterial-liver perfusion (ALP; mL/100 mL/min), portal-venous perfusion (PVP; mL/100 mL/min) and hepatic-perfusion index (HPI; %) using the maximum-slope model. ALP and PVP are calculated by taking the dual blood supply of the liver by the hepatic artery and portal vein into account. The time of peak splenic enhancement is used to separate the arterial- and portal-venous phase using ROIs in the portal vein and spleen. ALP is calculated by dividing the maximum arterial slope by the maximum aortic enhancement derived from the arterial time–density curve. PVP is calculated by dividing the maximum portal-venous slope by the maximum portal-vein enhancement derived from the portal-venous time–density curve. HPI represents the quotient from ALP and the sum of ALP and PVP.

All data were analyzed by three readers with 5, 9 and >20 years of experience in oncologic imaging in a consensus reading.

### 2.4. Chemoembolization

Catheter angiography of the hepatic and mesenteric artery was performed to evaluate liver and tumor vascular anatomy and for detection of possible arteriovenous shunts. Interventions were performed by an experienced interventional radiologist at the lowest possible radiation dose [23]. Feeding arteries were supra-selectively catheterized with a microcatheter (2.0-French Progreat α Terumo, Europe N.V, Leuven, Belgium). DC beads loaded with Epirubicin (BTG, Langweid/Augsburg, Germany) with a diameter of 100–300 µm were used with an average dose of 26.7 mg ± 13.5, range 10–75 mg. Beads were mixed with an equal volume of non-ionic contrast medium before delivery.

### 2.5. Statistical Analyses

Statistics were calculated with Prism (GraphPad 8 software, La Jolla, CA, USA). All data are reported as mean ± standard deviation (SD). Normal distribution was not given in the study data (D’Agostino & Pearson omnibus normality test, *p* < 0.05 for all datasets) and Mann–Whitney test or Kruskal–Wallis test were used for comparisons between groups. *p* values were corrected for multiple tests, and values smaller than 0.025 were considered significant.

## 3. Results

### 3.1. Patient Characteristics

Patients underwent perfusion CT 2.5 ± 4.6 days before TACE and always within 48 h thereafter. The initial lesion size was 30.1 ± 11.6 mm. Patient characteristics are presented in Table 1.

From the included 52 patients, 42 showed successful treatment 48 h after TACE (Figure 1) with no residual enhancement according to visual inspection of the perfusion maps and quantification in perfusion CT, which is in agreement with the interventional report.

**Figure 2:**
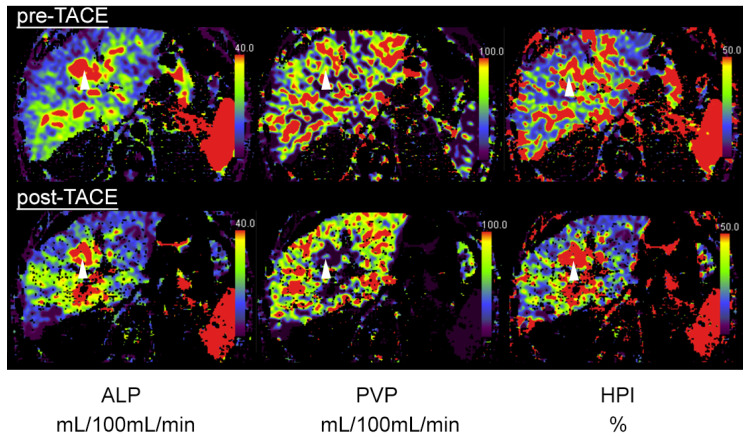
A 64-year-old patient with central HCC and incomplete embolization after transarterial chemoembolization (TACE). Pre-TACE images (top row) show increased arterial-liver perfusion (ALP, left, white arrowhead), slightly reduced portal-venous perfusion (PVP, **middle**) and increased hepatic-perfusion index (HPI, **right**) compared to normal liver tissue. Post-TACE images (bottom row) reveal a slight decrease in ALP (**left**), indicative of residual-tumor tissue.

In 10 lesions, only a partial tumor embolization was achieved according to the interventional report and subsequent perfusion CT (example in Figure 2).

**Figure 3:**
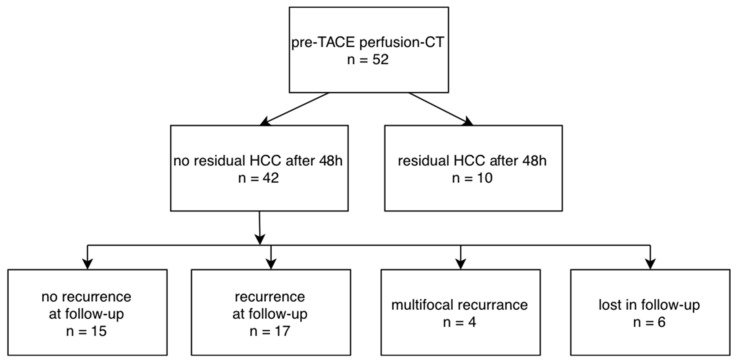
Flow-chart for included patients, results after TACE and follow-up.

Follow-up was performed in the 42 patients with no initial residual edge enhancement after TACE 95.3 ± 12.5 days thereafter, and 15 cases demonstrated no tumor recurrence and correspondingly no edge or core enhancement, whereas 17 cases showed recurring enhancement in the primarily embolized TACE area. Four patients showed multifocal and diffusely infiltrating recurrence and were excluded from analysis, as in these cases the lesions were not easily definable and could not be directly compared to the baseline lesion intended to be treated. Six patients did not show up at our institution for reassessment after three months (Figure 3).

### 3.2. Perfusion-CT Data

No significant differences were observed in uninvolved liver parenchyma, neither between baseline and post-TACE CT nor follow-up. Values are given in Table 2.

Quantitative analysis of perfusion parameters in HCCs before treatment showed an average ALP of 44.7 ± 15.0 mL/100 mL/min. PVP and HPI are additionally summarized in Table 2.

Patients with successful TACE showed reduced ALP values of the TACE/HCC region with 4.4 ± 5.3 mL/100 mL/min, which were significantly lower compared to non-responders (34.7 ± 10.1 mL/100 mL/min, *p* < 0.0001).

The rim of the TACE region was analyzed separately. Here, we found values resembling the TACE region (ALP responder 8.8 ± 8.7 mL/100 mL/min, non-responder 23.4 ± 8.6 mL/100 mL/min, *p* = 0.005). Compared to normal liver tissue after TACE, successfully treated tumors had lower values at the tumor margin compared to both normal liver tissue (mean rank difference −25.03) and non-responders (−12.76), while non-responders and normal liver tissue did not show significant differences (+12.27; Kruskal–Wallis statistic 12.31, *p* = 0.0021).

After an average of three months (95.3 ± 12.5 days after therapy), follow-up examinations with perfusion CT revealed local HCC recurrence in 17 patients with ALP values of 39.1 ± 10.1 mL/100 mL/min (Table 3). A total of 15 patients had no signs of recurrence (ALP 10.0 ± 7.4 mL/100 mL/min, *p* = 0.0008). The rim of the TACE region in patients with recurrence showed ALP values of 29.2 ± 6.3 mL/100 mL/min compared to 13.1 ± 10.0 mL/100 mL/min in patients with no recurrence (*p* = 0.083, n.s.). Values for ALP at the rim of the TACE region were significantly lower compared to the TACE region in recurring HCC (rim: 29.2 ± 6.3 mL/100 mL/min; recurring HCC: 39.1 ± 10.1 mL/100 mL/min, *p* = 0.014). In comparison, this was not true for non-recurring HCC (rim: 13.1 ± 10.0 mL/100 mL/min; TACE region, no recurrence: 10.1 ± 7.4 mL/100 mL/min, *p* = 0.80, n.s.).

## 4. Discussion

This study examined imaging findings in and around HCC treated with TACE by using PCT very early after TACE (within 48 h) and at mid-term (three months), focusing on a possible occurrence of rim enhancement in initially successfully embolized HCCs as a potential mimic of viable tumor mass or reactive hyperemia. Additionally, PCT maps were analyzed at the edges of the embolized tumor area for mid-term HCC relapse. PCT has a broad spectrum of applications, including characterization of liver lesions, especially in cirrhosis, as well as early response assessment for anti-VEGF therapy of HCC [24,25,26,27,28,29,30]. As PCT is also suitable for assessing response to TACE, we hypothesized that this modality might also be suitable to differentiate very early responses to TACE and residual hyperemia after the intervention. In contrast to MRI and CT studies performed several weeks after intervention, we did not observe any significant rim enhancement, neither shortly after TACE nor at mid-term follow-up. The tumor-free margins of the embolization areas were found to be even less arterially supplied compared to the normal liver parenchyma. This presumably reflects the impact of local transarterial embolization on the perilesional arterial supply of normal liver parenchyma following particle dispersion or embolization of marginal tumor areas. Previous reports stated that rim enhancement following local therapy for liver tumors could be a potential mimic of a residual tumor [6]. Guo et al. pointed at potential difficulties when assessing early response to intra-arterial therapies as related to reactive edema or granulation tissue formation [11]. Other studies emphasize that only a smooth homogenous rim should be visual, and that any nodular aspect should raise the suspicion of viable tumor tissue [6,7]. An inflammatory reaction to the thermal injury has been implicated in the transitory ring-like enhancement after RFA, whereas in SIRT patients the ring enhancement was found to correlate well with complete pathologic necrosis [12,13]. Following TACE, transient hyperemia has been considered a physiologic response to embolization of liver parenchyma surrounding the tumor itself. Chung et al. found 24% ring enhancement in HCCs treated with TACE, of which 83% proved to be benign at mid-term follow-up [6]. Their study design differed, as the follow-up CT was performed one month after TACE and the imaging technique used for perfusion assessment was a three-phase abdominal-CT protocol.

In our series, we examined the local effects of TACE within 48 h post-TACE and found no single case of ring enhancement with PCT. Moreover, we used a liver-perfusion protocol, which is expected to be more sensitive in detecting residual-tumor vascularization or reactive peritumoral hyperemia. To account for the lack of peripheral ring enhancement, we quantified the perfusion in a rim of liver parenchyma surrounding the embolization area, calculating the degree of arterial supply. Perfusion parameters knowingly differentiate between liver parenchyma and the tumor due to their different blood supply (arterial vs. portal venous) with HPI representing the percentage of arterial- to portal-venous supply in liver tissue and tumors.

Changes after interventions can be highly variable and misleading irrespective of progressive or responding disease. To address these issues, the use of standardized assessment criteria for follow-up imaging and case assessment by expert radiologists in interventional and oncological radiology is essential [31,32]. In our series, 42 HCCs showed complete embolization at the end of their respective TACE sessions and the following PCT revealed no evidence of residual-tumor enhancement or reactive ring enhancement in these cases. Hence, according to our results, TACE seems to have no imminent stimulatory impact on the perfusion of surrounding liver parenchyma—at least at this early time point—thus excluding reactive hyperemia as a potential differential for a persisting viable tumor after TACE. Furthermore, classifying ring enhancement as reactive hyperemia seems misplaced as there was no increase in perilesional perfusion at any time point, also whilst accounting for physiological intra-liver fluctuations. Another possible explanation for rim enhancement could be subsequent tissue scaring at the margins of the embolization area occurring later at follow-up.

Unfortunately, immediate post-TACE results are no guarantee of a relapse-free survival. Using CECT for post-RFA evaluation, Lu et al. reported a sensitivity of CT for the depiction of viable residual-tumor tissue of only 36% [33]. This is confirmed in recent studies from Müller et al. and Fronda et al. investigating the delayed percentage attenuation ratio from three- or four-phase CT and found this parameter helpful to determine early response [34,35]. However, similar to our results, this was not predictive for mid-term recurrence in the study of Müller et al. Conversely, peripheral, nodular, highly perfused areas of incomplete embolization always indicated early relapse due to viable tumor tissue at the margins after TACE. According to our results, they exhibited similar perfusion values as the pre-TACE tumor tissue or locally relapsed HCCs. As a potential future perspective, the possibilities for post-processing features such as convolutional neural networks, the pre-processed perfusion maps may present an easier dataset for artificial networks to operate on compared to the anatomical CT datasets, potentially allowing for predictions of disease recurrence based on baseline imaging alone or additional inclusion of post-TACE images. This might be a possible future integration of the PCT protocol to further implement it in clinical routine processes as previously described for other indications [36,37].

While PCT comes with an additional radiation burden and thus the indication must be critically validated, it has some advantages over MRI for specific cases. In addition to the use in patients with contra-indications for MRI, PCT can have advantages in cases with pronounced perihepatic ascites, where MRI artifacts can lead to improper liver signal. Compared to MRI, PCT examinations are relatively short with benefits for patients with reduced compliance and difficulties for breath hold examinations. Additionally, attempts to further reduce the PCT radiation dose are important [28,38,39]. This might be especially relevant in attempts to implement improved surveillance programs in several countries to identify early and even very early stages of HCC [40] to overcome the proposed limitations of ultrasound screening [41]. The identification of multiple characteristic small lesions with PCT or MRI could thus increase the demand for TACE in unresectable situations. As the vascular architecture and blood supply of very small lesions is substantially different from larger lesions, the presence of rim enhancement might be different compared to the investigated cohort. Future investigations of these phenomena will be of increasing relevance not only for patients treated with TACE but also for SIRT, where post-interventional response assessment might be even more complex, and a tendency towards response or failure of treatment might not be reliably accessible with imaging until several months after treatment [42].

Our study has some limitations. First, our cohort was small and therefore our results may not reflect the definite absence of a rim-enhancement phenomenon with PCT at the chosen time points. Second, the measured absolute values at the edges of tumor-embolization areas might have been influenced to some minor degree by partial volume averaging of the normal liver tissue or even the necrosis core despite robust motion-correction algorithms. Third, a possible limitation might be the fact that in this cohort, 21/42 patients showed recurrent enhancement at interim follow-up, which is suggestive of a residual or recurrent tumor. Despite this number, we still did not encounter cases of rim enhancement and unfortunately, this is not uncommon among TACE patients after the first treatment.

## 5. Conclusions

No case of rim enhancement was observed surrounding the embolized tumor area immediately after successful TACE or at mid-term follow-up. Our study indicates that perilesional rim enhancement is not a sign of reactive hyperemia as no increased perfusion was found. Unfortunately, the lack of peripheral enhancement proved to be no guarantee of a relapse-free mid-term follow-up.

## Figures and Tables

**Table 1:** Patient characteristics.

Number of patients (male/female)	52 (40/12)
Child–Pugh score	A (32), B (20)
BCLC Stage	B (52)
Underlying etiology	alcohol abuse (29), HCV (18), NASH (3), unknown (2)
Number of lesions	1–4
HCC tumor size	30.1 ± 11.6 mm

BCLC = Barcelona Clinic Liver Cancer staging system, HCV = Hepatitis C Virus, NASH = non-alcoholic steatohepatitis.

**Table 2:** Perfusion-CT data, baseline and post-TACE.

		Mean	SD	Minimum	Maximum	
Liver parenchyma (*n* = 52)	ALP	13.4	6.8	3.0	30.9	mL/100 mL/min
	PVP	60.3	25.2	23.3	137.6	mL/100 mL/min
	HPI	25.2	16.1	4.4	61.8	%
Liver parenchyma	ALP	16.3	10.1	1.9	48.9	mL/100 mL/min
post-TACE (*n* = 52)	PVP	62.3	26.6	16.9	112.6	mL/100 mL/min
	HPI	27.1	19.8	2.7	65.1	%
Liver parenchyma	ALP	9.0	12.4	0.9	38.4	mL/100 mL/min
follow-up (*n* = 23)	PVP	62.5	26.7	22.5	129.5	mL/100 mL/min
	HPI	25.4	25.0	1.4	73.1	%
HCC pre-TACE (*n* = 52)	ALP	44.7	15.0	14.3	101.6	mL/100 mL/min
	PVP	12.3	16.3	0.0	72.3	mL/100 mL/min
	HPI	85.8	16.6	18.2	100	%
HCC post-TACE,	ALP	4.4	5.3	0.0	21.5	mL/100 mL/min
responder (*n* = 42)	PVP	32.0	27.7	1.4	102.0	mL/100 mL/min
	HPI	30.4	26.8	0.0	83.4	%
HCC post-TACE,	ALP	34.7	10.1	20.6	43.2	mL/100 mL/min
non-responder (*n* = 10)	PVP	18.8	14.1	4.1	40.6	mL/100 mL/min
	HPI	67.7	21.4	39.8	93.4	%
Post-TACE rim region,	ALP	8.8	8.7	0.8	37.5	mL/100 mL/min
responder (*n* = 42)	PVP	49.2	42.4	4.0	186.3	mL/100 mL/min
	HPI	31.1	26.5	0.8	89.0	%
Post-TACE rim region,	ALP	23.4	8.6	12.3	33.2	mL/100 mL/min
non-responder (*n* = 10)	PVP	53.2	16.9	34.5	70.9	mL/100 mL/min
	HPI	33.0	7.0	27.0	42.6	%

SD = standard deviation, ALP = arterial-liver perfusion, PVP = portal-venous perfusion, HPI = hepatic-perfusion index.

**Table 3:** Perfusion-CT data, follow-up in initial responders.

		Mean	SD	Minimum	Maximum	
Follow-up TACE region,	ALP	10.0	7.4	3.0	19.9	mL/100 mL/min
no recurrence (*n* = 15)	PVP	37.5	5.3	33.5	45.2	mL/100 mL/min
	HPI	41.3	15.6	20.1	54.9	%
Follow-up TACE region,	ALP	39.1	10.1	22.3	61.4	mL/100 mL/min
recurrence (*n* = 17)	PVP	12.4	13.3	0.2	49.7	mL/100 mL/min
	HPI	85.6	15.4	50.4	99.3	%
Follow-up rim region,	ALP	13.1	10.0	6.0	20.2	mL/100 mL/min
no recurrence (*n* = 15)	PVP	39.4	34.7	14.9	63.9	mL/100 mL/min
	HPI	47.0	54.7	8.3	85.7	%
Follow-up rim region,	ALP	29.2	6.3	20.2	38.8	mL/100 mL/min
recurrence (*n* = 17)	PVP	22.3	13.1	5.8	45.6	mL/100 mL/min
	HPI	72.7	17.5	38.1	93.2	%

SD = standard deviation, ALP = arterial-liver perfusion, PVP = portal-venous perfusion, HPI = hepatic-perfusion index.

## Data Availability

Not applicable.

## References

[1] Llovet J., Brú C., Bruix J. (1999). Prognosis of Hepatocellular Carcinoma: The BCLC Staging Classification. Semin. Liver Dis..

[2] European Association for the Study of the Liver (2018). EASL Clinical Practice Guidelines: Management of hepatocellular carcinoma. J. Hepatol..

[3] Piñero F., Poniachik J., Ridruejo E., Silva M. (2018). Hepatocellular carcinoma in Latin America: Diagnosis and treatment challenges. World J. Gastroenterol..

[4] Agnello F., Salvaggio G., Cabibbo G., Maida M., Lagalla R., Midiri M., Brancatelli G. (2013). Imaging appearance of treated hepatocellular carcinoma. World J. Hepatol..

[5] Mendiratta-Lala M., Masch W.R., Shampain K., Zhang A., Jo A.S., Moorman S., Aslam A., Maturen K.E., Davenport M.S. (2020). MRI Assessment of Hepatocellular Carcinoma after Local-Regional Therapy: A Comprehensive Review. Radiol. Imaging Cancer.

[6] Chung W.-S., Lee K.-H., Park M.-S., Lee Y.J., Kwon J., Baek S.-E., Kim M.-J. (2012). Enhancement Patterns of Hepatocellular Carcinoma after Transarterial Chemoembolization Using Drug-Eluting Beads on Arterial Phase CT Images: A Pilot Retrospective Study. Am. J. Roentgenol..

[7] Kim S.K., Lim H.K., Kim Y.H., Lee W.J., Lee S.J., Kim S.H., Lim J.H., Kim S.A. (2003). Hepatocellular Carcinoma Treated with Radio-frequency Ablation: Spectrum of Imaging Findings. RadioGraphics.

[8] Kim Y., Rhim H., Lim H.K. (2009). Imaging After Radiofrequency Ablation of Hepatic Tumors. Semin. Ultrasound CT MRI.

[9] Riaz A., Kulik L., Lewandowski R.J., Ryu R.K., Giakoumis Spear G., Mulcahy M.F., Abecassis M., Baker T., Gates V., Nayar R. (2008). Radiologic-pathologic correlation of hepatocellular carcinoma treated with internal radiation using yttrium-90 microspheres. Hepatology.

[10] Baur J., Ritter C.O., Germer C.-T., Klein I., Kickuth R., Steger U. (2016). Transarterial chemoembolization with drug-eluting beads versus conventional transarterial chemoembolization in locally advanced hepatocellular carcinoma. Hepatic Med..

[11] Guo Y., Yaghmai V., Salem R., Lewandowski R.J., Nikolaidis P., Larson A.C., Miller F.H. (2013). Imaging tumor response following liver-directed intra-arterial therapy. Abdom. Imaging.

[12] Kallini J.R., Miller F.H., Gabr A., Salem R., Lewandowski R.J. (2016). Hepatic imaging following intra-arterial embolotherapy. Abdom. Radiol..

[13] Kumar Y., Sharma P., Bhatt N., Hooda K. (2016). Transarterial Therapies for Hepatocellular Carcinoma: A Comprehensive Review with Current Updates and Future Directions. Asian Pac. J. Cancer Prev..

[14] Syha R., Grözinger G., Grosse U., Maurer M., Zender L., Horger M., Nikolaou K., Ketelsen D. (2016). Parenchymal Blood Volume Assessed by C-Arm–Based Computed Tomography in Immediate Posttreatment Evaluation of Drug-Eluting Bead Transarterial Chemoembolization in Hepatocellular Carcinoma. Investig. Radiol..

[15] Yaghmai V., Besa C., Kim E., Gatlin J.L., Siddiqui N.A., Taouli B. (2013). Imaging Assessment of Hepatocellular Carcinoma Response to Locoregional and Systemic Therapy. Am. J. Roentgenol..

[16] Zou J.H., Zhang L., Ren Z.G., Ye S.L. (2016). Efficacy and safety of cTACEversusDEB-TACE in patients with hepatocellular carcinoma: A meta-analysis. J. Dig. Dis..

[17] Ippolito D., Fior D., Bonaffini P.A., Capraro C., Leni D., Corso R., Sironi S. (2014). Quantitative evaluation of CT-perfusion map as indicator of tumor response to transarterial chemoembolization and radiofrequency ablation in HCC patients. Eur. J. Radiol..

[18] Ippolito D., Bonaffini P.-A., Ratti L., Antolini L., Corso R., Fazio F., Sironi S. (2010). Hepatocellular carcinoma treated with transarterial chemoembolization: Dynamic perfusion-CT in the assessment of residual tumor. World J. Gastroenterol..

[19] Kaufmann S., Horger T., Oelker A., Beck S., Schulze M., Nikolaou K., Ketelsen D., Horger M. (2015). Volume perfusion computed tomography (VPCT)—Based evaluation of response to TACE using two different sized drug eluting beads in patients with nonresectable hepatocellular carcinoma: Impact on tumor and liver parenchymal vascularisation. Eur. J. Radiol..

[20] Ronot M., Lambert S., Daire J.-L., Lagadec M., Doblas S., Garteiser P., Kerbaol A., Sinkus R., Van Beers B.E., Vilgrain V. (2013). Can we justify not doing liver perfusion imaging in 2013?. Diagn. Interv. Imaging.

[21] (2012). EASL–EORTC Clinical Practice Guidelines: Management of hepatocellular carcinoma. J. Hepatol..

[22] Chandler A., Wei W., Anderson E.F., Herron D.H., Ye Z., Ng C.S. (2012). Validation of motion correction techniques for liver CT perfusion studies. Br. J. Radiol..

[23] Compagnone G., Giampalma E., Domenichelli S., Renzulli M., Golfieri R. (2012). Calculation of conversion factors for effective dose for various interventional radiology procedures. Med. Phys..

[24] Kurucay M., Kloth C., Kaufmann S., Nikolaou K., Bösmüller H., Horger M., Thaiss W.M. (2017). Multiparametric imaging for detection and characterization of hepatocellular carcinoma using gadoxetic acid-enhanced MRI and perfusion-CT: Which parameters work best?. Cancer Imaging.

[25] Thaiss W.M., Kaufmann S., Kloth C., Nikolaou K., Bösmüller H., Horger M. (2016). VEGFR-2 expression in HCC, dysplastic and regenerative liver nodules, and correlation with pre-biopsy Dynamic Contrast Enhanced CT. Eur. J. Radiol..

[26] Kaufmann S., Horger T., Oelker A., Kloth C., Nikolaou K., Schulze M., Horger M. (2015). Characterization of hepatocellular carcinoma (HCC) lesions using a novel CT-based volume perfusion (VPCT) technique. Eur. J. Radiol..

[27] Kaufmann S., Thaiss W.M., Schulze M., Bitzer M., Lauer U., Nikolaou K., Horger M. (2017). Prognostic value of perfusion CT in hepatocellular carcinoma treatment with sorafenib: Comparison with mRECIST in longitudinal follow-up. Acta Radiol..

[28] Ippolito D., Sironi S., Pozzi M., Antolini L., Ratti L., Alberzoni C., Leone E.B., Meloni F., Valsecchi M.G., Fazio F. (2008). Hepatocellular Carcinoma in Cirrhotic Liver Disease. Acad. Radiol..

[29] Ippolito D., Querques G., Okolicsanyi S., Talei Franzesi C., Pecorelli A., Lombardi S., Orsini E., Strazzabosco M., Sironi S. (2018). Dynamic contrast enhanced perfusion CT imaging: A diagnostic biomarker tool for survival prediction of tumour response to antiangiogenetic treatment in patients with advanced HCC lesions. Eur. J. Radiol..

[30] Ippolito D., Querques G., Pecorelli A., Talei Franzesi C., Okolicsanyi S., Strazzabosco M., Sironi S. (2019). Diagnostic Value of Quantitative Perfusion Computed Tomography Technique in the Assessment of Tumor Response to Sorafenib in Patients with Advanced Hepatocellular Carcinoma. J. Comput. Assist. Tomogr..

[31] Lencioni R., Montal R., Torres F., Park J.-W., Decaens T., Raoul J.-L., Kudo M., Chang C., Ríos J., Boige V. (2017). Objective response by mRECIST as a predictor and potential surrogate end-point of overall survival in advanced HCC. J. Hepatol..

[32] Tovoli F., Renzulli M., Negrini G., Brocchi S., Ferrarini A., Andreone A., Benevento F., Golfieri R., Morselli-Labate A.M., Mastroroberto M. (2018). Inter-operator variability and source of errors in tumour response assessment for hepatocellular carcinoma treated with sorafenib. Eur. Radiol..

[33] Lu D.S.K., Yu N.C., Raman S.S., Limanond P., Lassman C., Murray K., Tong M.J., Amado R.G., Busuttil R.W. (2005). Radiofrequency Ablation of Hepatocellular Carcinoma: Treatment Success as Defined by Histologic Examination of the Explanted Liver. Radiology.

[34] Müller L., Hahn F., Jungmann F., Mähringer-Kunz A., Stoehr F., Halfmann M.C., Pinto Dos Santos D., Hinrichs J., Auer T.A., Düber C. (2022). Quantitative washout in patients with hepatocellular carcinoma undergoing TACE: An imaging biomarker for predicting prognosis?. Cancer Imaging.

[35] Fronda M., Doriguzzi Breatta A., Gatti M., Calandri M., Maglia C., Bergamasco L., Righi D., Faletti R., Fonio P. (2021). Quantitative assessment of HCC wash-out on CT is a predictor of early complete response to TACE. Eur. Radiol..

[36] Paul R., Shafiq-Ul Hassan M., Moros E.G., Gillies R.J., Hall L.O., Goldgof D.B. (2020). Deep Feature Stability Analysis Using CT Images of a Physical Phantom across Scanner Manufacturers, Cartridges, Pixel Sizes, and Slice Thickness. Tomography.

[37] Paul R., Hawkins S.H., Balagurunathan Y., Schabath M.B., Gillies R.J., Hall L.O., Goldgof D.B. (2016). Deep Feature Transfer Learning in Combination with Traditional Features Predicts Survival among Patients with Lung Adenocarcinoma. Tomography.

[38] Enjilela E., Lee T.-Y., Hsieh J., Murjoomdar A., Stewart E., Dekaban M., Su F., So A. (2017). Ultra-Low-Dose Sparse-View Quantitative CT Liver Perfusion Imaging. Tomography.

[39] Kalarakis G., Perisinakis K., Akoumianakis E., Karageorgiou I., Hatzidakis A. (2021). CT liver perfusion in patients with hepatocellular carcinoma: Can we modify acquisition protocol to reduce patient exposure?. Eur. Radiol..

[40] Park H.J., Jang H.Y., Kim S.Y., Lee S.J., Won H.J., Byun J.H., Choi S.H., Lee S.S., An J., Lim Y.-S. (2020). Non-enhanced magnetic resonance imaging as a surveillance tool for hepatocellular carcinoma: Comparison with ultrasound. J. Hepatol..

[41] Tzartzeva K., Obi J., Rich N.E., Parikh N.D., Marrero J.A., Yopp A., Waljee A.K., Singal A.G. (2018). Surveillance Imaging and Alpha Fetoprotein for Early Detection of Hepatocellular Carcinoma in Patients With Cirrhosis: A Meta-analysis. Gastroenterology.

[42] Levillain H., Bagni O., Deroose C.M., Dieudonné A., Gnesin S., Grosser O.S., Kappadath S.C., Kennedy A., Kokabi N., Liu D.M. (2021). International recommendations for personalised selective internal radiation therapy of primary and metastatic liver diseases with yttrium-90 resin microspheres. Eur. J. Nucl. Med. Mol. Imaging.

